# A comparative study of the efficacy of Chinese herbal medicine Duhuo Jisheng decoction combined with DMARDs vs isolated DMARDs for rheumatoid arthritis: A protocol for systematic review and meta analysis

**DOI:** 10.1097/MD.0000000000023479

**Published:** 2020-12-11

**Authors:** Xing Zhou, Kemeng Xiang, Minyuan Lu, Hanting Xia, Xingchen Zhou, Xiangyao Yuan, Zhenping Wang, Kuanglin Li

**Affiliations:** a Taizhou Traditional Chinese Medicine Hospital, Taizhou City, Zhejiang Province; b The First Clinical College, Fujian University of Traditional Chinese Medicine; c Fuzhou Traditional Chinese Medicine Hospital, Fuzhou City, Fujian Province; d Affiliated Hospital of Jiangxi University of Traditional Chinese Medicine, Nanchang City, Jiangxi Province, China.

**Keywords:** disease-modifying anti-rheumatic drugs, Duhuo Jisheng decoction, protocol, randomized controlled trial, rheumatoid arthritis, systematic review

## Abstract

**Background:**

Rheumatoid arthritis (RA) is a chronic autoimmune system disease that mainly affects joints throughout the body, causing joint pain, deformity, and even disability. The use of Chinese herbal medicine (CHM) to treat RA has achieved certain effects, and Duohuo Jisheng decoction (DHJSD) is one of them. But there is no high-level evidence to support this result. The purpose of this work is to evaluate the effectiveness of DHJSD combined with DMARDs compared with isolated DMARDs for RA.

**Methods:**

We will search articles in 7 electronic databases including Chinese National Knowledge Infrastructure (CNKI), Wanfang Data (WF), Chinese Scientific Journals Database (VIP), Chinese databases SinoMed (CBM), PubMed, Embase, and Cochrane Library databases. All the publications, with no time restrictions, will be searched without any restriction of language and status, the time from the establishment of the database to October 2020. Two reviewers will independently assess the quality of the selected studies, NoteExpress and Excel software will be used to extract data, and the content will be stored in an electronic chart. Different researchers will separately screen the titles and abstracts of records acquired potential eligibility which comes from the electronic databases. Full-text screening and data extraction will be conducted afterward independently. Statistical analysis will be conducted using RevMan 5.4 software.

**Results:**

This study will evaluate the efficacy and safety of DHJSD combined with DMARDs compared with isolated DMARDs in the treatment of Rheumatoid arthritis, to provide high-quality, evidence-based clinical recommendations.

**Conclusion:**

This study will provide reliable evidence on whether Duhuo Jisheng decoction combined with DMARDs compared with isolated DMARDs is more effective in treating RA.

**Trial registration number:**

INPLASY2020100089.

## 1 Introduction

Rheumatoid arthritis (RA) is a chronic systemic autoimmune disease,^[1]^ that mainly invades and destroys synovial joints, causing joint stiffness, pain, and swelling in the early stage.^[2]^ In severe cases, it can lead to disability, reduced life expectancy.^[3]^ Studies have shown that worldwide, the incidence of RA is between 0.4% and 1.3%.^[4,5]^ and bring a heavy burden to individuals and society. Therefore, it is very urgent to seek a safe and effective treatment for RA, which can continuously relieve the clinical symptoms of patients with morning stiffness, pain, and swelling, fundamentally inhibit the progressive damage of tissues and joints, and delay or prevent the development of the disease.

The specific pathogenesis of RA is not clear, but it is closely related to genetic factors, among which human leukocyte antigen (HLA) genes occupy a major position in causing autoimmune diseases.^[6]^ Synovial joint cells are the main target of autoimmunity in RA, T cells and B cells migrate into synovial joints and interact with macrophages, synoviocytes, osteoclasts, etc, to produce degrading enzymes and some inflammatory cytokines (such as interleukin [IL]-1, and IL-6), leading to the proliferation of synovial lining cells and massive inflammation of the interstitium sex cell infiltration, as well as the formation of microvessels and pannus, destroy cartilage and bone tissue. The clinical manifestations are joint stiffness, swelling, and pain.^[2]^

In terms of treatment options, drug control is a conventional method, including non-steroidal anti-inflammatory drugs (NSAIDs) , glucocorticoids, DMARDs and biological agents, etc.^[3]^ DMARDs are preferred and recommended used once the patient is diagnosed with RA.^[7,8]^ It is well known that DMARDs have side effects such as infection, liver damage, cytopenia, and poor tolerance (fatigue, nausea, and central nervous system side-effects), etc.^[9,10]^ but they still need to be taken for a long time or even for life. The therapeutic effect of a single anti-rheumatic drug is sometimes not ideal. Combining other western drugs may aggravate side effects or increase the incidence of adverse reactions.

The treatment of RA with traditional Chinese medicine (TCM) has received more and more attention,^[11]^ and the treatment method of integrated traditional and western medicine has obtained satisfactory clinical results.^[12]^ Duhuo Jisheng decoction (DHJSD) is derived from Simiao Sun, a famous Chinese medicine practitioner, it is composed of 15 traditional Chinese medicines: Doubleteeth Pubescent Angelica Root, Chinese Taxillus Twig, Largeleaf Gentian Root, Divaricate Saposhnikovia Root, Manchurian Wild Ginger, Szechwan Lovage Rhizome, Angelica Root, Rehmannia, White Peony Root, Cinnamon Bark, Sclerotium of Tuckahoe, Eucommia Bark, Achyranthes Root, Ginseng Root, and Licorice root.^[13,14]^ That is widely used to treat RA, Modern pharmacological studies have shown that DHJSD can play an anti-inflammatory effect by inhibiting the expression of TNF-α, IL, and CRP.^[15]^ Animal experiments show that DHJSD can also alleviate cartilage degeneration in CIA rats by regulating the number of mitochondria and Golgi, up-regulating the expression of serum osteoprotegerin (OPG), and increasing OPG/NF-κB receptor activator ligand (RANKL). Delay the differentiation and maturation of osteoclasts, thereby effectively delaying RA bone erosion.^[16,17]^ Since DHJSD has a good anti-inflammatory and anti-rheumatic mechanism and has achieved certain clinical effects, it is worthy of further exploration in adjuvant combined DMARDs therapy. However, currently, there is no higher-level evidence-based medical evidence to systematically evaluate and analyze the safety and efficacy of DHJSD combined with DMARDs in the treatment of RA. Therefore, this work aims to achieve the above-mentioned goals through systematic reviews and meta-analysis, and provide reliable evidence for the clinic.

## 2 Methods

### 2.1 Study registration

This protocol report is structured according to the Preferred Reporting Items for Systematic Reviews and Meta-analysis Protocols (PRISMA-P) statement.^[18]^ It is registered on the International Prospective Register of Systematic Reviews. (Registration number INPLASY2020100089; https://inplasy.com/inplasy-2020-10-0089/).

### 2.2 Inclusion criteria

#### 2.2.1 Type of study

Only randomized controlled trials will be included irrespective of blinding, publication status, or language in this study.

#### 2.2.2 Types of participants

Patients were diagnosed with rheumatoid arthritis and the study belongs to randomized controlled trial. Clinical results included clinical effectiveness, Rheumatoid factor (RF), Erythrocyte sedimentation rate (ESR), C-reactive protein (CRP), symptom evaluation (including morning stiffness, pain, and joint swelling), and adverse effects. The experimental group must cover Duhuo Jisheng decoction combined with Disease-modifying anti-rheumatic drugs (DMARDs) and the control group must cover Disease-modifying anti-rheumatic drugs (DMARDs). Otherwise, studies will be excluded if they cannot meet the inclusion criteria.

#### 2.2.3 Types of Interventions

The intervention of the experimental group must cover Duhuo Jisheng decoction (DHJSD) combined with Disease-modifying anti-rheumatic drugs (DMARDs). There are no restrictions on the way of dosage and treatment period.

#### 2.2.4 Types of control groups

The control group must cover Disease-modifying anti-rheumatic drugs (DMARDs).

#### 2.2.5 Outcomes

##### 2.2.5.1 Primary outcome measures

The primary outcome is symptom evaluation (including the number of swelling joints affected by RA; the number of painful joints affected by RA; the duration of morning stiffness).

##### 2.2.5.2 Secondary outcomes

The secondary outcome are clinical effectiveness, Rheumatoid factor (RF), Erythrocyte sedimentation rate (ESR), C-reactive protein (CRP) and adverse effects.

### 2.3 Search strategy

CNKI, Wanfang, VIP, CBM, PubMed, Embase, and Cochrane Library databases were searched for this study. Take the subject terms combined with free words to search, take PubMed as an example: terms consist of disease (Arthritis, Rheumatoid OR Rheumatoid Arthritis) and intervention (Duhuo Jisheng decoction OR Duhuo Jisheng Tang) and Comparison (Antirheumatic Agents OR DMARDs OR Methotrexate OR Sulfasalazine OR Leflunomide OR Iguratimod) and research types (randomized controlled trial OR controlled clinical trial OR random trials) as shown in Table 1.

**Table 1:** Pubmed database search strategy.

Search number	Items
1	“Arthritis, Rheumatoid”[Mesh]
2	Rheumatoid Arthritis [Title/Abstract]
3	1 OR 2
4	Duhuo Jisheng decoction [Title/Abstract]
5	Duhuo Jisheng Tang [Title/Abstract]
6	4 OR 5
7	“Antirheumatic Agents”[Mesh]
8	DMARDs [Title/Abstract]
9	Methotrexate [Title/Abstract]
10	Sulfasalazine [Title/Abstract]
11	Leflunomide [Title/Abstract]
12	Iguratimod [Title/Abstract]
13	7 OR 8 OR 9 OR 10 OR 11 OR 12
14	randomized controlled trial [Title/Abstract]
15	controlled clinical trial [Title/Abstract]
16	random trials [Title/Abstract]
17	14 OR 15 OR 16
18	3 AND 6 AND 13 AND 17

### 2.4 Data collection and analysis

#### 2.4.1 Selection of studies

**Figure 1:**
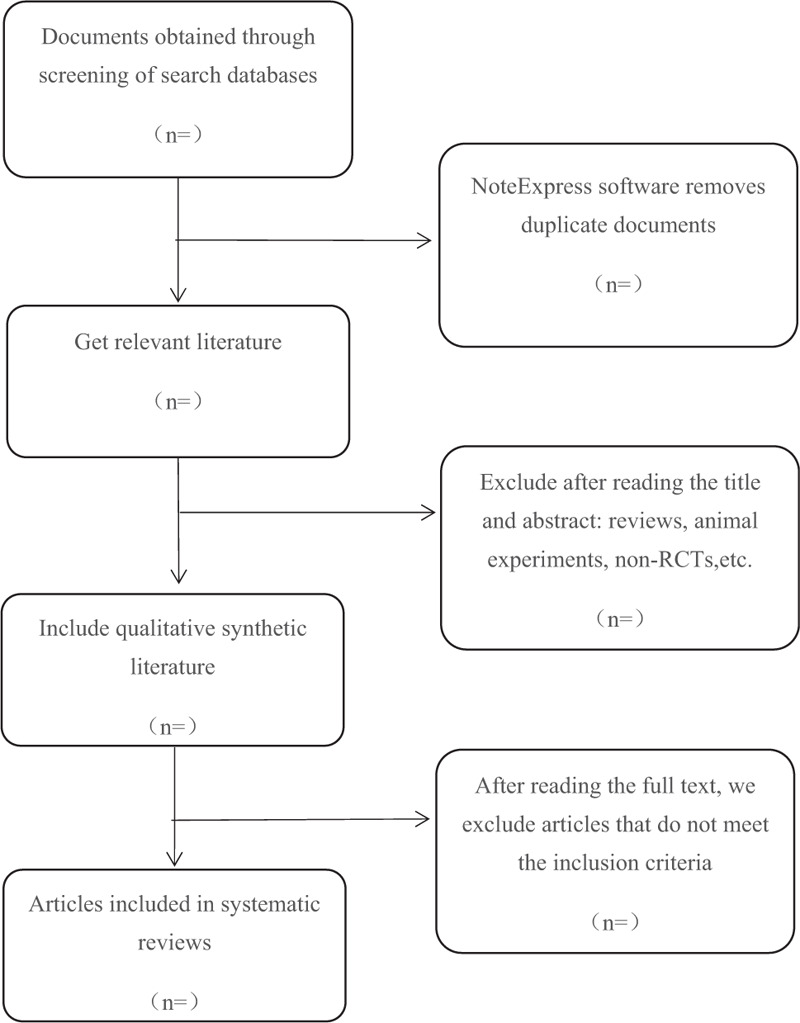
Flowchart of literature selection.

Different researchers will separately screen the titles and abstracts of records acquired potential eligibility which comes from the electronic databases. The obtained literature is managed by Notoexpress, irrelevant and duplicate articles are excluded by reading the title and abstract, full texts screening and data extraction will be conducted afterward independently, and finally selected according to the inclusion criteria, any disagreement will be resolved by discussion until consensus is reached or by consulting a third author. PRISMA-P flowchart will be used to show the selection procedure (Fig. 1).

#### 2.4.2 Data extraction and management

NoteExpress and Excel software will be used to extract data, and the content will be stored in an electronic chart. The following data will be extracted: author, year of publication, country, interventions of experimental groups and control groups, time point, outcome measures, age of patients, the total number of people included in the study, patients basic information, etc. Different researchers will separately extract data. Any disagreement regarding data extraction will be resolved by discussion until consensus is reached or by consulting a third author.

### 2.5 Risk of bias assessment

Two reviewers will independently assess the quality of the selected studies according to the Cochrane Collaboration's tool for randomized controlled trials.^[20]^ Items will be evaluated in 3 categories: Low risk of bias, unclear bias, and high risk of bias. The following characteristics will be evaluated: random sequence generation (selection bias), allocation concealment (selection bias), blinding of participants and personnel (performance bias), incomplete outcome data (attrition bias), selective reporting (reporting bias), and other biases. Results from these questions will be graphed and assessed using Review Manager 5.4. The results will be presented in the form of a graph and will be independently evaluated by 2 researchers. If there are differences of opinion, they will be discussed with the third researcher

### 2.6 Statistical analysis

Statistical analysis will be conducted using RevMan 5.4 software. For continuous data, will be used mean difference (MD) as the effect indicator with 95% confidence interval, and dichotomous data will be calculated as risk ratio (RR) or odds ratio (OR) as the effect index with 95% confidence interval. The *I*^2^ statistic will be used to assess levels of heterogeneity, when *I*^2^ < 50%, the fixed-effect model can be used for analysis, otherwise, the random-effect model will be used.

### 2.7 Sensitivity analysis

Through sensitivity analysis assess the source of heterogeneity, by excluding low-quality studies, or by excluding one of the included studies in turn, if there is no significant change in the heterogeneity, the results are robust, otherwise, the excluded study may be the heterogeneous originate.

### 2.8 Subgroup analysis

We will consider subgroups analysis the course of the disease, and the intervention time.

### 2.9 Publication bias

In this study, less than ten RCTs will use funnel plots to evaluate publication bias, or else, Egger's regression test will be used.

## 3 Discussion

DHJSD is a classic TCM prescription. It is recommended for use in the 2018 Chinese RA Disease and Syndrome Combination Guidelines.^[19]^ Due to its good anti-inflammatory, antioxidant, and bone destruction effects, it has been clinically verified in the treatment of RA and has fewer adverse reactions. The drug treatment program of DHJSD combined with DMARDs aims to better improve and relieve the condition of patients with RA. However, there is still a lack of higher-level evidence-based medicine evidence to support this choice. Therefore, this study is to provide a more credible basis for future clinicians to make decisions.

This study still has certain shortcomings, because some factors will lead to biased results, such as low-quality original research, the intervention period, etc, which will weaken the reliability of the evidence.

## Author contributions

**Conceptualization:** Xing Zhou, Kemeng Xiang, Minyuan Lu.

**Data curation:** Xing Zhou, Kemeng Xiang, Minyuan Lu, Hanting Xia, Xingchen Zhou, Xiangyao Yuan, Zhenping Wang, Kuanglin Li.

**Formal analysis:** Xingchen Zhou, Xiangyao Yuan, Kuanglin Li.

**Investigation:** Hanting Xia, Zhenping Wang, Kuanglin Li.

**Methodology:** Xing Zhou, Kemeng Xiang, Xingchen Zhou.

**Software:** Kemeng Xiang, Xingchen Zhou.

**Supervision:** Hanting Xia, Xingchen Zhou.

**Writing – original draft:** Xing Zhou, Minyuan Lu.

**Writing – review & editing:** Minyuan Lu, Hanting Xia, Xingchen Zhou, Zhenping Wang.
